# Temporal dynamics of Puumala hantavirus infection in cyclic populations of bank voles

**DOI:** 10.1038/srep21323

**Published:** 2016-02-18

**Authors:** Liina Voutilainen, Eva R. Kallio, Jukka Niemimaa, Olli Vapalahti, Heikki Henttonen

**Affiliations:** 1 Natural Resources Institute Finland, Vantaa, Finland; 2 University of Helsinki, Department of Virology, Finland; 3 University of Jyvaskyla, Department of Biological and Environmental Science, Finland; 4 Helsinki University Hospital, Department of Virology, Finland

## Abstract

Understanding the dynamics of zoonotic pathogens in their reservoir host populations is a prerequisite for predicting and preventing human disease epidemics. The human infection risk of Puumala hantavirus (PUUV) is highest in northern Europe, where populations of the rodent host (bank vole, *Myodes glareolus*) undergo cyclic fluctuations. We conducted a 7-year capture-mark-recapture study to monitor seasonal and multiannual patterns of the PUUV infection rate in bank vole populations exhibiting a 3-year density cycle. Infected bank voles were most abundant in mid-winter months during years of increasing or peak host density. Prevalence of PUUV infection in bank voles exhibited a regular, seasonal pattern reflecting the annual population turnover and accumulation of infections within each year cohort. In autumn, the PUUV transmission rate tracked increasing host abundance, suggesting a density-dependent transmission. However, prevalence of PUUV infection was similar during the increase and peak years of the density cycle despite a twofold difference in host density. This may result from the high proportion of individuals carrying maternal antibodies constraining transmission during the cycle peak years. Our exceptionally intensive and long-term dataset provides a solid basis on which to develop models to predict the dynamic public health threat posed by PUUV in northern Europe.

Understanding the dynamics of zoonotic pathogens in their reservoir host populations is an important step towards predicting the risk of zoonotic diseases to humans, such as infections caused by hantaviruses that account for ca. 50 000 cases worldwide each year^1^,^2^. Hantaviruses (family *Bunyaviridae*) are carried by rodents, soricomorphs, and bats^3^. Some of them cause haemorrhagic fever with renal syndrome (HFRS; viruses harboured by Old World rats and mice, as well as arvicolines) or hantavirus cardio pulmonary syndrome (HCPS; viruses harboured by New World rats and mice) in humans. In terms of human health, the most important European avirus is Puumala hantavirus (PUUV) which causes nephropathia epidemica (NE), a mild form of HFRS^4^,^5^. The principal host of PUUV is the bank vole (*Myodes glareolus*), which is distributed over most of Europe^6^ and is the dominant rodent species in boreal forests.

In general, the incidence of human hantavirus infections tracks the abundance of the local rodent host population^7^,^8^,^9^,^10^,^11^,^12^. In temperate Europe, bank vole populations show seasonal fluctuations and occasional, irregular eruptions caused by masting, i.e., a heavy seed crop of oak and beech^12^,^13^,^14^. Masting has been linked to human NE epidemics, likely brought about by growth of the local vole population^12^,^15^,^16^. In boreal forests of northern Europe, deciduous trees do not undergo masting events and vole population abundance cycles of 3 to 5 years are thought to be driven by specialist predators^17^,^18^,^19^. Across Europe, the incidence of NE is highest in Finland, northern Sweden and northern Russia^5^,^20^. Although bank vole population dynamics have proven useful for predicting NE epidemics in both temperate and boreal biomes^9^,^10^,^11^,^12^,^21^, detailed analyses of long-term PUUV transmission in reservoir populations are lacking. Furthermore, the relationship between rodent abundance and human infection rate is somewhat obscure; for example, a higher incidence of human NE was observed during increasing rather than peak phases in two vole cycles in central Finland where PUUV is endemic^9^.

Our objective was to fully describe the pattern of PUUV transmission in cyclic populations of boreal bank voles. More specifically, we aimed to characterize the seasonal and multiannual fluctuations in 1) the abundance of PUUV-infected bank voles, which is presumably the main driver of NE epidemics in humans, 2) the prevalence of PUUV infection, and 3) the rate of PUUV transmission (as indicated by the acquisition rate of PUUV-specific antibodies), which we expected to shed more light on within-host transmission of PUUV. In addition, we studied whether the accumulation or rate of PUUV infections differed between annual cohorts born in different cycle phases. For these purposes, we monitored PUUV infections in bank voles through nearly three vole cycles using a capture-mark-recapture method. For the first time, PUUV infection dynamics were also studied in detail during the winter when the incidence of human NE is highest in the boreal zone.

## Methods

### Ethical statement

All handling procedures of wild bank voles followed the Finnish Act on the Use of Animals for Experimental Purposes (62/2006) and took place with permission from the Finnish Animal Experiment Board (license numbers HY 45-02, HY 122-03, and HY 54-05). All efforts were made to minimize animal suffering. The species studied is not protected in Finland or included in the Red List of Finnish Species. The animal trapping took place with permission from the landowners.

### Rodent trapping and sampling

The study took place at Konnevesi in central Finland (62°34′ N, 26°24′ E), where PUUV is highly endemic in bank voles^9^,^22^,^23^,^24^. In this region, the ground is covered by snow for an average of 160 days from late November to late April^25^. PUUV transmission dynamics were studied in detail using a capture-mark-recapture (CMR) method on a large “core grid”. Additionally, trappings were performed on 14 smaller “satellite grids” to extend the geographic scale of the dataset. The core grid included a young stand of birch and willow on drained soil surrounded by mature spruce-dominated coniferous forest with the understorey vegetation dominated by mosses and dwarf shrubs (e.g., *Vaccinium*). Satellite grids were situated in pine, spruce, and mixed forests of different ages within 5 km of the core grid and at least 750 m from it and each other. Trappings took place from April 2002 to May 2009.

The core grid consisted of 246 trapping stations at 15 m intervals, covering a total area of 5.8 ha. One Ugglan Special live-trap (Grahnab, Sweden) was placed at each trapping station under a sheet metal chimney that provided shelter for captured animals but enabled access to traps during the snowy period. In all, 60 trapping sessions were conducted on the core grid. During the snow-free period, the trapping interval was approximately one month and longer during winters, so that the intervals lasted from 17 to 166 days (median 37, interquartile range 30–43 days). Traps were baited with oat seeds and potato and set in the evening. After setting, traps were checked ten times at ca. 8-hour intervals. During low temperatures (< –5 °C) in winter, traps were checked 3 times per day at 4-hour intervals and left open (non-trapping) overnight. Newly-trapped bank voles were subcutaneously tagged with a transponder (ID-100A Microtransponder, Trovan Ltd, U.K.). On each trapping session, voles were bled through the retro-orbital sinus and their body mass, age (based on pelage^26^), sex, and sexual maturity (perforate and/or lactating females and males with scrotal testes regarded as mature) were recorded. Animals recaptured during a single trapping session were immediately released.

A total of 21 trapping sessions on the 14 satellite grids were conducted in the beginning (May), middle (July), and after the end (October) of the bank vole breeding season. Nine Ugglan Special live traps baited with oat seeds and potato were set in a grid of 3*3 traps with 15 m intervals for 3 nights and checked once per day. Occupied traps were replaced with freshly-baited ones and animals were brought into the laboratory, bled through retro-orbital sinus, and sacrificed. In October 2004 and July and October 2006, live traps were replaced with standard snap traps baited with rye bread. The sex and breeding status of all animals snap-trapped in the satellite grids were determined before the heart was dissected and placed into 200 μL phosphate-buffered saline and stored at –20 °C prior to the analysis of PUUV antibodies.

### Determination of PUUV infection status for each animal in each trapping session (t)

All blood (diluted 1:10 in phosphate-buffered saline) and heart samples were tested for the presence of PUUV-specific antibodies using an immunofluorescent antibody test (IFAT) described elsewhere^27^. Based on IFAT results, an infection status was determined for each animal for each trapping session (*t*). The classifications of infection status are shown in Table 1 (hereafter, uppercase letters indicate infection status deduced from the animal’s serological history, and lowercase letters indicate that the status was assigned according to other criteria).

As PUUV causes a chronic infection in the bank vole^28^, a PUUV-seropositive (P) result was interpreted as an infected (I) animal. However, young animals captured between May and October may be seropositive as a result of maternal antibodies (MatAb) received from an infected female which provide temporary immunity for up to 80 days from birth^29^. Therefore, initially seropositive young animals that were later captured and determined to be seronegative were considered as being MatAb + (M) when first captured. Determining the infection status of young animals that were caught in more than one trapping session and which always tested seropositive was more complex. For those seropositive animals captured in the core and satellite grids, the number genuinely infected (i) was estimated for each trapping session by summing the assigned individual probabilities (estimate based on body mass) of being genuinely infected. These probabilities were calculated from a statistical model (i.e., MatAb model, Supplementary Fig. S1; see below) based on 588 captures with a known serological history in the core area; i.e., 1) animals that had acquired or/and lost antibodies between the two trapping sessions, and 2) seropositive animals that were old enough (>8 months) to exclude the possibility of a positive result due to MatAb. In the MatAb model, infection status was examined in relation to body mass using generalized additive models (GAM) with a binomial distribution (0 = MatAb +, 1 = genuinely infected) and logit link function (gam function of gamm4 library^30^ in the R software package^31^). Because the growth rate of young animals varies over the breeding season depending on if they mature immediately or delay reproduction, separate models were run for each month of capture (Supplementary Fig. S1). If an animal was assigned a probability >0.9 of being genuinely infected, it was considered as genuinely infected (probability of being infected = 1) in later trapping sessions. For example, if a summer-born bank vole was first captured as seropositive in August weighing 19.4 grams, seropositive in September weighing 18.4 grams, and seropositive in October weighing 16.7 grams, it was allocated respective probabilities 0.924, 0.908, and 0.963 of being genuinely infected by the GAM models. However, as the initial probability (0.924) exceeded 0.9, the animal was assigned a probability of 1 of being infected, and infection status II (infected in *t – 1* and *t*), for September and October.

Seronegative animals were considered as non-infected and therefore susceptible (S). In some rare cases, an adult individual was seronegative despite being previously considered genuinely infected. These antibody results were interpreted as false negatives. When an animal was not captured during a particular trapping session (*t*), but captured with an unchanged PUUV antibody status between the preceding (*t – 1*) and the following (*t* + *1*) trapping sessions, that same antibody status was also determined for trapping session *t*.

### Determination of PUUV infection status in the previous trapping session (*t*–*1*) for each animal captured in trapping session t

To calculate the seroconversion rate (the number of individuals that acquired PUUV antibodies between trapping sessions *t – 1* and *t* out of those susceptible at *t – 1*) in the core grid, the infection status in the previous trapping session (*t – 1*) for each animal captured in trapping session *t* needed to be determined. The possible preceding (*t – 1*) status were: I (Infected), S (Susceptible), M (MatAb+), and 0 (suckling pups and thus not a part of the population). For animals that were captured and tested for antibodies at both *t – 1* and *t*, determination was straightforward. However, for individuals that were first time captured at *t*, probabilities of belonging to the susceptible group at the previous trapping session were assigned (see details in Table 1).

Firstly, young animals likely to have been suckling pups and therefore not part of the susceptible population at *t–1* were excluded from the analysis. The exclusion criterion was a body mass at *t* that was lower than the body mass of the lightest animal captured for the second time in the same month of any year.

Secondly, young individuals that were seronegative (?S) at their first capture (*t*) were divided into seronegative and therefore susceptible (sS) at *t – 1*, and seropositive due to maternal antibodies and therefore non-susceptible (mS) at *t – 1*: their assigned numbers were calculated using the proportion susceptible at *t–1* (SS) among the seronegative individuals at *t* (SS + MS), so that (sS) = (?S)*[SS/(MS + SS)], and (mS) = (?S) –(sS). In nine early summer trapping sessions, none of the young summer-born animals (N = 38) had a known serological history and therefore this calculation method could not be applied. In these cases, the proportion of individuals carrying MatAb at *t – 1* [mS/(sS + mS)] was determined to be the same as the infection prevalence among over-wintered females in the preceding trapping session (*t – 1*); according to earlier results, PUUV infection has no effect on breeding success in early summer among over-wintered individuals^29^,^32^.

Thirdly, for individuals that were first captured as seropositive, and from which the MatAb and 0 individuals at *t – 1* had been extracted, i.e., (?i) = (?P–0M–mM–0m–mm), the number that seroconverted between *t – 1* and *t* (si) was estimated using the proportion that seroconverted between *t – 1* and *t* (SI) among the known positive individuals (SI + II) so that (si) = (?i)*[SI/(SI + II)]. Especially during the breeding season, animals born in different years (i.e., old over-wintered and young summer-born voles) coexisted on the study grid. Given that different-age animals likely fall into the four infection classes (0, M, S, I) on divergent proportions, all the above-mentioned assignments were made per year cohort, i.e., animals born in the same summer.

### Statistical analyses

#### Data sets

The population-level dynamics of (a) the abundance of infected animals, (b) the prevalence of infection, and (c) the *per capita* seroconversion rate were examined using two population-level datasets (datasets “Prevalence of PUUV per trapping session” and “Seroconversion rate per trapping session”, available from the Dryad Digital Repository: http://dx.doi.org/10.5061/dryad.g8140 33). In addition to population-level dynamics, we examined the effects of age and cycle phase on the accumulation of infections and the seroconversion rate in yearly cohorts of bank voles using two cohort-level datasets (datasets “PUUV prevalence per year cohort” and “Seroconversion rate per year cohort”, available from the Dryad Digital Repository: http://dx.doi.org/10.5061/dryad.g8140 33).

The seasonal and multiannual variation in the abundance of infected individuals was studied using a dataset (“Prevalence of PUUV per trapping session”^33^) of trapping indices (individuals/100 trap nights) of PUUV-infected bank voles from all trapping sessions conducted on the core grid (60 trapping sessions, 246 traps) and the 14 satellite grids (21 trapping sessions, 126 traps). The same dataset was used to study the temporal variation in PUUV infection prevalence. For the satellite grids, infection prevalence was calculated as the number of individuals known to be (or assigned as) infected per the total number tested, and for the core grid as the minimum number of infected per the minimum population size (i.e., including animals that were not captured at *t* but at least once before and once after *t*).

The dataset used to study temporal variation in seroconversion rate (“Seroconversion rate per trapping session”^33^) consisted of the *per capita* seroconversion rate calculated for each trapping session on the core grid by dividing the total number of seroconverted by the total days of exposure, i.e., (SI + si)/[(SS + SI + ss + si)* interval], where (SI + si) was the number of animals known to be or scored as seroconverted between *t – 1* and *t*, (SS + SI + ss + si) was the number of animals known to be or scored as susceptible on *t – 1*, and “interval” was the number of days between *t – 1* and *t*. Individuals with MatAb were excluded from the susceptible population. According to population abundances observed here and in an earlier study^9^, the trapping sessions were divided into two categorical variables: three phases of the density cycle (variable “cycle phase”: “increase”, “peak”, and “low” phases; onset of phase on 1^st^ June) and three vole density cycles (variable “cycle ID”: 2001−2003, 2004−2006, and 2007−2009; onset of cycle on June 1^st^ of the increase phase). June was selected as the onset of a biological year, as the first summer-born individuals usually entered the population in that month. Also a dichotomous variable “breeding season” was included in the data set, indicating whether the time interval between *t – 1* and *t* predominantly belonged to the yearly breeding season or not.

In addition to studying infection prevalence and seroconversion rate in the whole population, we examined the effects of age and cycle phase on the accumulation of infections and the seroconversion rate in yearly cohorts of bank voles. Thus, infection prevalences (“PUUV prevalence per year cohort”^33^) and seroconversion rates (“Seroconversion rate per year cohort”^33^) were calculated for each birth year cohort and for each trapping session they were present on the core grid. Age was determined as the number of months after 1^st^ May of the year of birth, i.e., the earliest month when individuals of the year-born cohort could be expected to enter the population (although they were not observed in our trapping data). Birth years were divided into “increase”, “peak”, and “low” phases as above.

#### Candidate models

The population-level dynamics of (a) the abundance of infected animals, (b) the prevalence of infection, and (c) the *per capita* seroconversion rate (datasets “Prevalence of PUUV per trapping session” and “Seroconversion rate per trapping session”^33^) were examined within two time-frames: a biological year and a three-year population density cycle. In yearly cohorts of bank voles (datasets “PUUV prevalence per year cohort” and “Seroconversion rate per year cohort”^33^), the accumulation of PUUV infections and the temporal variation in seroconversion rate were studied in a time-frame of 16 months, the assumed maximum life span of most bank voles^34^. Generalized additive models (GAM, gamm4 package^30^ for R software^31^) were applied in all analyses that included continuous temporal variables (i.e. “cycle month”, “month”, and “cohort age”). GAMs are nonparametric regression models often used for characterizing associations where the shape of the relationship between the predictors and the response is not known *a priori* – for instance, in temporal variation^35^. Generalized additive mixed models (GAMMs, function “gamm4”), i.e. GAMs including random effects, were used for population-level analyses on the abundance of infected individuals and infection prevalence (dataset “Prevalence of PUUV per trapping session”^33^), since the dataset included observations from several sites (core and 14 satellites). Models without random effects (function “gam”) were used for other analyses. The abundance of infected individuals was analysed using a log link function with Poisson error distributions. The trapping effort (number of trap nights) was used as an offset variable. The prevalence of infection and seroconversion rate (proportional outcomes) were analysed using a logit link function with binomial error distributions, weighted by the total number of individuals and exposure time (N susceptible individuals*interval between *t – 1* and *t*), respectively. Overdispersion was accounted for by including an observation-level random effect in mixed models, and a dispersion parameter in models without random effects.

For the population-level dynamics (i.e., abundance of infected animals, infection prevalence and the *per capita* seroconversion rate in the total population), a set of candidate models was examined for each time-frame (see Table 2). The “within-cycle” candidate model set included a full model consisting of factorial variable “cycle ID” and separate smooth terms of months elapsed (i.e. “cycle month”) from the cycle onset (1^st^ of June) for each “cycle ID”. The full model of the “within-year” model set included the factorial variable “cycle phase” and separate smooth terms of time elapsed from 1^st^ June (“month”) for each “cycle phase” level (Table 2). All submodels nested within the full models were included in the candidate model sets.

Seasonal variation in seroconversion rate was further studied using the dichotomous variable “breeding season” in dataset “Seroconversion rate per trapping session”^33^ as a temporal predictor instead of a continuous time variable. The full model of the candidate model set (“Variation in relation to breeding season”, Table 2) was a generalized linear model with binomial error distributions and a logit link function, where the interaction between cycle phase and breeding season was set as an explanatory variable.

In yearly cohorts of bank voles, the accumulation of PUUV infections and the temporal variation in seroconversion rate were studied with a candidate model set that included a full model consisting of the factorial variable “Birth year cycle phase” and separate smooth terms of cohort age (from 1^st^ May) for each cycle phase, and all submodels nested within the full model (model set “Variation in relation to cohort age and birth year cycle phase”, Table 2).

All candidate model sets were ranked according to Akaike information criteria adjusted for sample size (AICc^36^, in MuMIn package^37^ for R software^31^). In model sets where dispersion parameters were used to account for overdispersion, quasi-AICc (QAICc) was used for model ranking^38^. In each candidate model set, the most parsimonious model within 2 AICc/QAICc units from the lowest AICc/QAICc score was considered the best supported by the data and subsequently used for statistical inference. For mixed model sets, an optimal random effects structure was chosen for each full model in a similar fashion prior to the selection of fixed effects, so that models with random variance attributable to a) trapping site b) trapping site for each factor level (“cycle ID” or “cycle phase”), and c) observation (to account for overdispersion), and combinations a + b and a + c were ranked according to AICc scores. In all analyses, P values below 0.05 were considered statistically significant.

## Results

**Figure 1:**
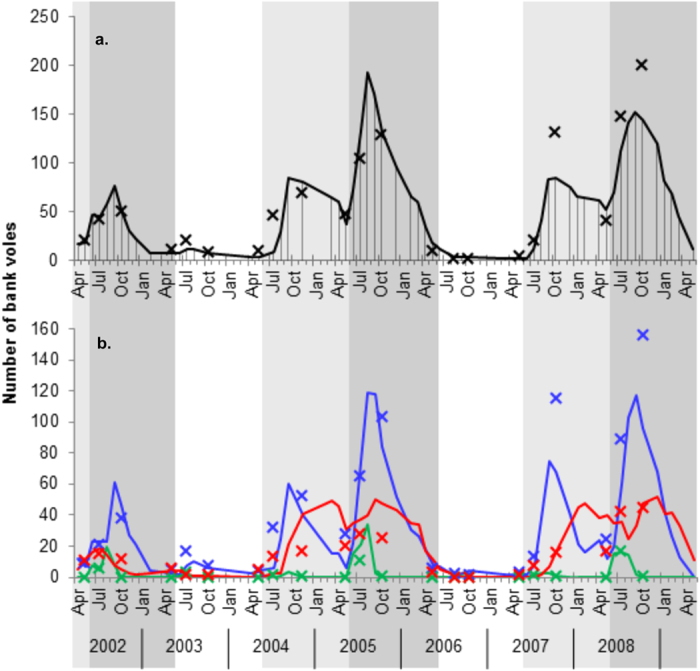
The number of bank voles on the core (lines) and satellite grids (crosses), (**a)** in total and (**b**) according to PUUV infection status. Values for the 14 satellite grids are pooled. Shaded areas indicate phases (Jun 1^st^ to May 31^st^) of increasing (light grey), peak (dark grey) and low (white) vole density. In a, vertical lines indicate trapping sessions on the core grid. In b, colours indicate infection status: susceptible for PUUV infection (blue), infected with PUUV (red), and MatAb+ (green).

During the 7-year study, bank vole abundance exhibited a distinct three-year cycle in which the population increased, peaked and then crashed (Fig. 1a). During the increase and peak phases, the highest abundances were reached at the end of the breeding season (August-October) while abundance remained low during the same period in low phases.

A total of 1 108 bank voles were captured on the core grid, of which 615 (56%) were captured in at least two trapping sessions. At first capture, 337 individuals were PUUV seropositive, 766 were seronegative, and the antibody status could not be determined for five animals. The median number of captures per animal during separate trapping sessions was 2 (interquartile range 1−4), and the maximum number of trapping sessions the same individual was captured was 14. In total, 3 286 captures (i.e., individuals known to be alive at a given trapping session *t* but for which an infection status could not be determined) were obtained: 1 415 (43%) testing PUUV seropositive and 1 871 (57%) seronegative (Table 1). A total of 1 152 bank voles were caught on the satellite grids, of which 324 (28%) were PUUV seropositive, 790 (69%) seronegative and 38 (3%) could not be tested.

Of the 766 initially-seronegative voles caught on the core grid, 499 were recaptured at least once during following trapping sessions, yielding a total of 1 240 recaptures where an animal’s infection status was SS or SI (Table 1). Among these recaptures of susceptible animals, 223 seroconversions (18%) were recorded, of which 35% took place between the first and second capture, 31% between the second and third capture, and the remaining 34% between the third and fourteenth capture. Of the 337 initially-seropositive animals, 236 were first captured in their birth year between May and October and potentially, due to their young age, carried MatAb. Of these 236, 126 were recaptured at least once and 78 (62%) became seronegative and were thus considered to have been carrying MatAb. For the 200 captures of the remaining 158 initially-seropositive first-year animals, the assigned probabilities (calculated for each capture from the MatAb model based on body mass, see Supplementary Fig. S1) of being infected summed to 98.4, meaning that a total of 200 − 98.4 = 101.6 captures (51%, 0m + mm in Table 1) were considered MatAb positive. MatAb+ animals were more common in peak phase cohorts than those born in other cycle phases and accounted for approximately 25–35% of seropositive animals in July of peak phases (Fig. 1b). The number of susceptible, infected, and MatAb+ animals on the core grid were similar to pooled numbers of animals caught on the satellite grids (Fig. 1b).

### Temporal patterns in PUUV infection

#### Abundance of infected voles

**Figure 2:**
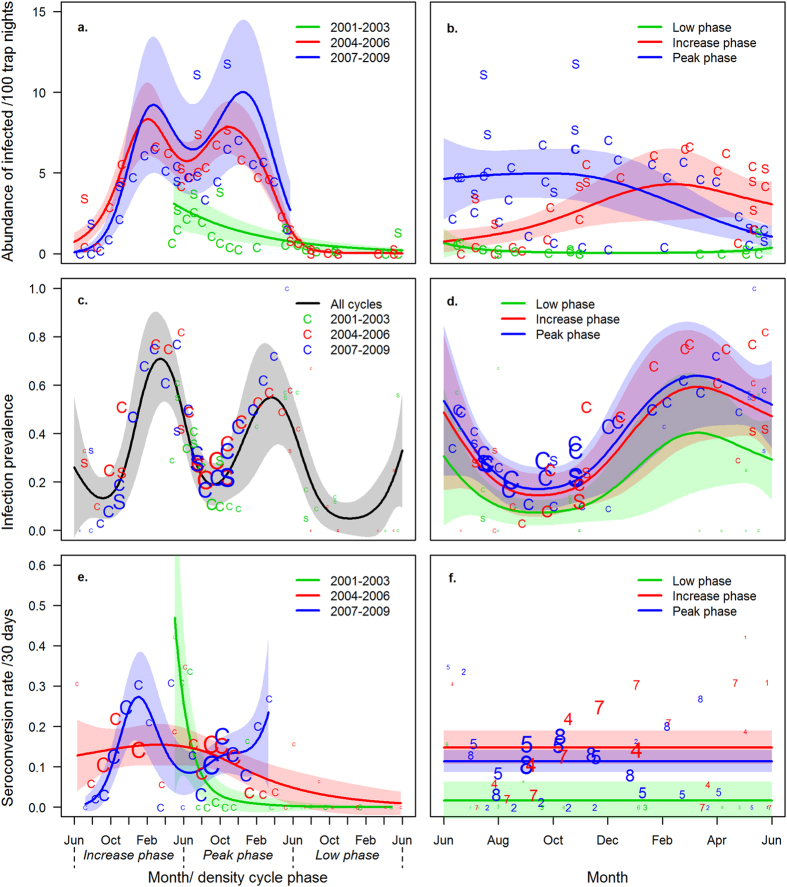
Variation in the (**a,b**) abundance of PUUV-infected bank voles/100 trap nights, (**c,d**) PUUV infection prevalence, and (**e,f**) seroconversion rate within the time frame of (**a,c,e**) a vole density cycle and (**b,d,f**) a year. Lines represent predicted values for the core grid from the best-supported models ((a) cycle ID + s[cycle month by cycle ID]; (b) cycle phase + s[month by cycle phase]; (c) s[cycle month]; (d) cycle phase + s[month]; (e) cycle ID + s[cycle month by cycle ID]; (f) cycle phase; s[…] denote GAM smooth terms). Shaded areas indicate the 95% confidence intervals of parameter estimates. “C” and “S” in a−e denote observed data in core and pooled satellite grids, respectively. Numbers 1−8 in f denote biological years 2001−2008. The character sizes indicate the number of animals (**c,d**) and total days of exposure (**e,f**). Green, red, and blue colours indicate different cycles in (**a,c,e**) and different cycle phases in (**b,d,f**).

On the timescale of a three-year density cycle, the dynamics in the abundance of PUUV-infected bank voles on the core and satellite grids were best explained by a GAMM that included the factorial variable “cycle ID” and separate smooth terms of time elapsed from the cycle onset for each cycle (“cycle month”, Tables 2 and 3). According to the model, infection patterns during density cycles 2004–2006 and 2007–2009 were similar, when the abundance of infected voles reached high peaks during both increase- and peak-phase winters (Fig. 2a, Table 3). On the core grid during increase phases, the peak abundance of infected individuals was 8.3 (95% confidence interval [CI]: 6.1–10.6) and 9.2 (95% CI: 5.0–13.5) infected individuals/100 trap nights during the 2004–2006 and 2007–2009 cycles, respectively, and occurred in February. In peak phases, 7.8 (95% CI: 6.2–9.5) and 10.0 (95% CI: 5.5–14.5) infected individuals/100 trap nights were attained in late October and early December in 2004–2006 and 2007–2009 cycles, respectively. During the increase phases of these cycles, the peak abundance of infected voles followed the peak in total abundance with a lag of approximately 3.5 to 4.5 months, whereas during peak phases, the lag was only 2 months (Figs 1a and 2a). The 2001–2003 cycle (data from April 2002 to June 2003) differed from the other two cycles in that the abundance of infected voles remained much lower and peaked during the summer of the peak phase with 3.1 (95% CI: 1.6–4.7) individuals/100 trap nights, then declined towards winter (Fig. 2a).

Within a time frame of one year, the abundance of infected animals was best explained by a model that included the factorial variable “cycle phase” and separate smooth terms for temporal variation (“month”) in each cycle phase (Tables 2 and 3). During increase phases, a seasonal pattern could be seen with the abundance of infected animals increasing from 0.3 (95% CI: 0.1–0.8) in June to 4.3 (95% CI: 2.1–6.5) in early February, and thereafter declining to 3.1 (95% CI: 1.7–4.5) individuals/100 trap nights by the end of May (Fig. 2b). During peak phases, the model suggested a rather stable level of infection from June to late November (4.6–5.0 infected individuals/100 trap nights, 95% CI: 2.1–7.2) thereafter declining to 1.1 individuals (95% CI: 0.4–1.7) by the end of May. During low phases, the predicted abundance of infected animals was nearly constant and remained below one individual/100 trap nights (Fig. 2b).

#### Infection prevalence

The prevalence of PUUV infection on the core and satellite grids showed a clear seasonal fluctuation both within cycles (Fig. 2c) and within years (Fig. 2d). Models with distinct temporal patterns for separate cycles or separate cycle phases were not supported (Table 2). The best supported within-cycle model only included the temporal component, i.e., a smooth term for the time elapsed from cycle onset, and the best within-year model included the seasonal component, i.e., a smooth term of time elapsed since the onset of the biological year and separate intercepts for cycle phases (Table 3). According to the within-cycle model, the PUUV prevalence peaked in mid-March of the increase phase (prevalence 69.3%, 95% CI: 49.9–88.7%), and a second, but lower peak followed in mid-March of the peak phase (prevalence 52.7%, 95% CI: 30.7–74.7%). Within a cycle, the lowest infection prevalences occurred in early September during both increasing and peak phases (PUUV prevalences 12.4% [95% CI: 4.1–20.7%] and 17.8% [95% CI: 9.1–26.5%], respectively), and in early December during low phase (prevalence 4.2%, 95% CI: 0–10.2%; Fig. 2c). The within-year model predicted a yearly prevalence peak in mid-March, when 59% (95% CI: 39–83%), 64% (95% CI: 44–86%), and 40% (95% CI: 20–65%) of bank voles were infected during increase, peak and low phases of the cycle, respectively (Fig. 2d). The yearly bottom prevalence was predicted to occur in mid-September, when 15% (95% CI: 6–23%), 17% (95% CI: 8–26%), and 7% (95% CI: 2–13%) of animals were infected during increase, peak and low phases, respectively. In the within-year model, there was no significant difference between increase and peak phase prevalences, but during low phases the prevalence was significantly lower than during increase phases (Table 3).

#### Per capita seroconversion rate

From the within-cycle model set examining the *per capita* seroconversion rate, the best-supported model included separate smooth terms of time for different cycles (Tables 2 and 3), and the patterns of seroconversion rate showed no similarity between cycles (Fig. 2e). According to model predictions for cycle 2007−2009, the seroconversion rate increased from July to January in increase phase 2007 and from August to October in peak phase 2008. In the within-year model set, the best-supported model included separate intercepts for cycle phases and no within-year temporal variation, although there was no significant difference between cycle phases (Tables 2 and 3, Fig. 2f). Although within-year models with temporal components were not well supported (Table 2), an increasing trend in seroconversion rate was observed from August to October in increase phases 2004 and 2007, and peak phases 2005 and 2008 (Fig. 2f). This increase was captured in within-cycle model predictions for years 2007 and 2008 (cycle 2007−2009 in Fig. 2e). In the model set where the continuous time variables were replaced with the dichotomous variable “breeding season”, the model “breeding season + cycle phase” was best supported (Table 2), so that the seroconversion rate was significantly higher outside than during the breeding season (coefficient for non-breeding season = 0.39, SE = 0.18, z = 2.1, P = 0.036).

### PUUV infection prevalence and seroconversion rate in relation to cohort age and cycle phase at birth

**Figure 3:**
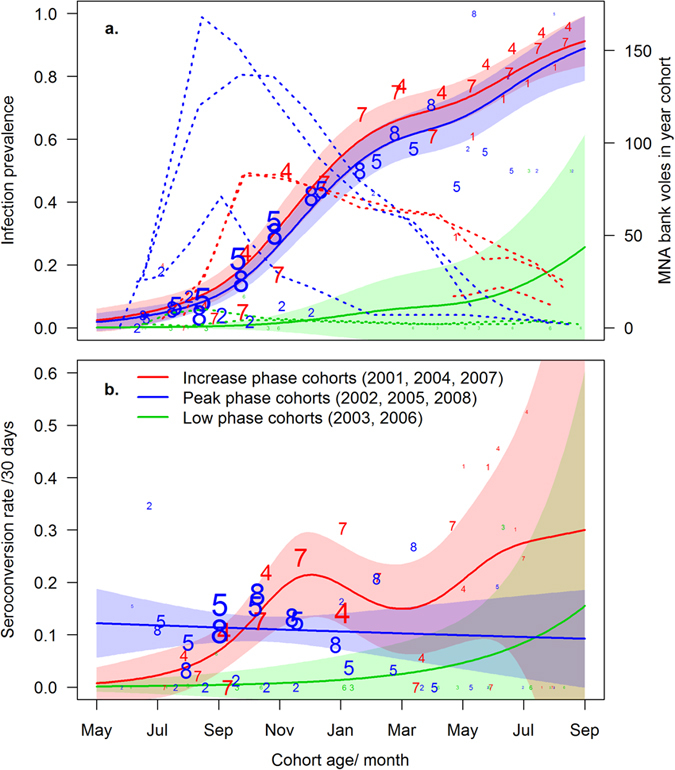
The (**a**) prevalence of PUUV infection and (**a**) seroconversion rate during the lifespan of a yearly bank vole cohort in relation to density cycle phase at birth. Solid lines represent predicted values from the best-supported models ((**a**) cycle phase + s[cohort age]; (b) cycle phase + s[cohort age by cycle phase]; s[…] denote GAM smooth terms). Shaded areas indicate the 95% confidence intervals of parameter estimates. Dashed lines in a indicate the total number of individuals in yearly cohorts, and colours denote the cycle phase at the summer of birth. Numbers 1−8 denote yearly cohorts 2001 to 2008, their size indicating the total (**a**) and the number of susceptible (**b**) animals.

Of the model set examining infection prevalence in relation to age and cycle phase of the birth year, the best-supported model included a common smooth term of cohort age but separate intercepts for each birth year cycle phase (Tables 2 and 4). The gradual increase of infection prevalence with age was similar in increase- and peak-phase cohorts (Fig. 3a): in both cohorts, nearly all surviving individuals were PUUV infected by the end of their second summer (increase phases: 91.4%, 95% CI: 83.4–99.4%; peak phases: 89.1%, 95% CI: 78.8–99.4%). In cohorts born during low phases, infection prevalence was significantly lower (Table 4), reaching only 27.4% (95% CI: 0.0–65.5%) by the end of their second summer. Overall, the infection prevalence increased steadily with cohort age, although a period of steeper increase was observed between September and January. The total number of animals reached its peak between August and October in every year cohort (Fig. 3a, dashed lines). However, the peak abundances of increase-phase cohorts (2004 and 2007: Fig. 3a dashed red lines) were 40–50% lower than those of peak-phase cohorts (2005 and 2008: Fig. 3a dashed blue lines).

Seroconversion rate between two trapping sessions in a year cohort was best explained by a model that included separate smooth terms of age for cohorts born in different cycle phases (Tables 2 and 4). The temporal patterns of predicted seroconversion rates were dissimilar (Fig. 3b): seroconversion rate showed a steady increase from September to December in increase-phase cohorts, resulting in a steep increase in infection prevalence (Fig. 3a). From December to March, the rate declined and increased thereafter coinciding with the onset of the breeding season and continuing into summer, although with wide confidence intervals. In peak-phase cohorts, no temporal trend was seen in seroconversion rate except for a slight, steady decline (Fig. 3b). However, seroconversion rates in peak-phase cohorts 2005 and 2008 exhibited similar patterns from July to January (Fig. 3b, blue numbers), and in four year cohorts (2004, 2005, 2007, and 2008) the seroconversion rate increased from August to October (Fig. 3b). Similar patterns were seen when yearly cohorts were combined for each trapping session (Fig. 2f). Low-phase cohorts showed a rather steady seroconversion rate in relation to cohort age, mainly remaining lower than those of other cycle-phase cohorts (Fig. 3b).

## Discussion

The abundance of PUUV-infected bank voles followed a similar temporal pattern from one host density cycle to another; over two consecutive 3-year cycles, infected animals were most abundant during winters of the increase and peak phases. Infection prevalence showed a seasonal pattern reflecting the age structure of the bank vole population; prevalence increased with age, peaking in spring when the population consists of old overwintered animals. The PUUV seroconversion rate showed irregular temporal patterns between density cycles, although an increasing trend was observed during the autumn in several years, and the rate was significantly higher outside the breeding season. The dynamics of PUUV and the bank vole population were very similar between the core and satellite grids, indicating that events documented in detail on the core grid represent patterns at a larger landscape level.

### Population-level temporal dynamics of PUUV infection

Our data from three density cycles demonstrated that the abundance of infected individuals followed strikingly similar temporal patterns through two pronounced cycles taking place 2004–2006 and 2007–2009. However, long-term datasets tracking vole population fluctuations in Finland indicate alternating periods of stronger and weaker cyclicity^39^, and a longer but less intensive time series (1995 to 2008) described how the bank vole population at Konnevesi showed a primarily seasonal pattern 1995–1998 but became increasingly cyclic 2001−2009^9^,^24^. In periods of less-prominent cyclicity, the abundance of infected voles may follow different seasonal patterns, as evidenced by the moderate peak phase 2002, when the maximum number of infected animals remained lower than in other peak phases and had already been reached in July. In general, the abundance peaks of infected voles followed the yearly peaks of total bank vole abundance, which likely results from a continuing spread of PUUV in the overwintering population. Interestingly, the abundance of infected voles during peak phases 2005 and 2008 did not exceed that of the preceding years (2004, 2007) in which the vole population was smaller.

Infection prevalence of bank voles in the core and pooled satellite grids showed a strongly seasonal pattern, peaking in spring, similar to what has been documented earlier for PUUV in boreal^9^,^24^,^40^,^41^ and temperate biomes^42^, as well as for other hantaviruses^43^. Interestingly, our intensive monitoring study showed that prevalence peaked in March, three months before the entry of the next cohort in June, implying that PUUV-infected voles faced a higher mortality during this period. This could be explained by the physiological stress associated with the onset of breeding or scarcity of resources prior to the onset of growth season in late April. The finding is in line with our earlier study where PUUV infection decreased the survival of bank voles between October and May^22^. Our current results suggest that survival costs of PUUV are related to breeding costs and/or food shortage in early spring. However, as our study design did not allow us to distinguish between death and dispersal, it is also possible that PUUV-infected voles performed poorly in territorial contests and were more likely to disperse to suboptimal habitats as a consequence.

Although bank voles were scarce during the low phases, the same yearly pattern of infection prevalence was supported by the within-year model. The within-cycle model, however, predicted lower prevalences for the low phases and no peak in early spring, which may result from scarce data: during low phases, trapping was not conducted in winter and only a few animals were captured in spring. Thus, high winter and spring prevalences may have been missed and we cannot conclude whether a particular host density — as suggested for PUUV in temperate bank vole populations^44^ and Sin Nombre virus in deer mouse populations of south-western United States^45^ — is required for PUUV to persist in a boreal bank vole population. Although not significant, infection prevalence was somewhat lower in the peak phases compared to the increase phases. This suggests that factors other than host density (discussed in the next paragraph) play a significant role in regulating the prevalence of PUUV infection in bank voles, which predominantly fluctuates seasonally according to the predictions of a within-year model.

No consistent temporal patterns of seroconversion rate were supported by the statistical analyses, which may be due to the noise associated with these data. When susceptible animals are scarce, the observed seroconversion rate is vulnerable to sampling error and the estimate may deviate considerably from the actual. In addition, variation in trapping intervals may have obscured any subtle patterns from being detected. For example, during the increase phase 2004, no trappings were conducted between October 2004 and April 2005 and the observed seroconversion rate between these trapping sessions (data point in January 2005) clearly deviates from the pattern observed in the winter of increase phase 2007 when trappings were more frequent (Fig. 2e&f and Fig 3b). However, when susceptible animals were abundant and trappings were performed more regularly (e.g., during the autumn of increase and peak phases 2004, 2005, 2007 and 2008), a clear increase in seroconversion rate from August to October could be seen. Furthermore, the overall seroconversion rate was higher outside than during the breeding season. An increase in seroconversion rate in the autumn and its high level during the winter suggests that susceptible animals become less resistant to infection or experience more frequent contact with infective individuals. The increasing seroconversion rate tracked the increasing population density and coincided with the increasing abundance of PUUV-infected bank voles, supporting the earlier view^46^ of hantavirus transmission being density dependent. However, harsh late autumn and winter conditions could impair physiological condition and immune investment^47^, thereby increasing the susceptibility of bank voles to infection as well as increase the viral shedding of infected animals^48^. Furthermore, PUUV particles remain infectious for longer in cold and humid conditions^49^ under the snowpack, and bank voles are not territorial outside the breeding season^50^. All these factors may not only increase the average abundance of infectious viral particles in the environment and the susceptibility of hosts, but could also increase exposure by bringing susceptible and infectious animals into close proximity, thus increasing the rate of PUUV transmission. It remains for future analyses to assess the extent to which the observed seasonal and multiannual patterns are attributable to host population dynamics – such as the density of susceptible and/or infected individuals – and whether the seasonal variation in environmental conditions, such as humidity, snow cover and temperature could influence the observed seasonal and multiannual dynamics of seroconversion rate. In contrast to studies in which aggression has been identified as a key driver of transmission for other hantaviruses in their host populations^51^,^52^,^53^, we found a higher PUUV transmission rate outside the breeding season when bank voles do not show aggressive behaviour. This finding suggests that aggression is not an important factor affecting PUUV transmission in boreal bank voles, and that the key routes of transmission may vary between hantavirus host systems.

### PUUV infection prevalence and seroconversion rate in year cohorts

In most hantavirus-rodent host systems studied, older or heavier individuals have been associated with a higher likelihood of being infected^24^,^41^,^42^,^54^,^55^. This pattern has been assumed to result from accumulated time of exposure, and to underlie the seasonal fluctuation of infection prevalence observed in seasonally-breeding host species^43^,^56^. Our current data corroborate this process in detail and demonstrate a steady increase in PUUV infection prevalence in yearly cohorts of bank voles, reaching virtually 100% during the cohort lifespan in high density phases. This finding emphasises the importance of host population demography as a determinant of infection prevalence, especially during the breeding season when individuals born in different years are present.

Compared to cohorts born in increase and peak phases, significantly fewer bank voles acquired PUUV infection in low-phase cohorts. However, no more than four animals born in low-phase summers were captured after the first autumn in any of the trapping sessions, and therefore the absence of infected voles may have been coincidental. Although the phase of steepest increase in total abundance occurred in peak-phase cohorts two months earlier than in increase-phase cohorts, the infection prevalence did not respond to the earlier increase but increased at the same pace in both cohorts. Likewise, during the autumn the *per capita* seroconversion rate in increase-phase cohorts 2004 and 2007 showed a similar pattern to that observed in peak-phase cohorts 2005 and 2008. These results imply that host abundance alone does not regulate the rate of PUUV infection in bank voles. However, a larger portion of peak-phase cohorts was protected by MatAb than in increase-phase cohorts, which agrees with an earlier study of voles in the same area^24^. The presence of MatAb+ individuals may have compensated for the effect of higher host density, so that PUUV infection and seroconversion rate remained unchanged between increase- and peak-phase cohorts despite the twofold difference in maximum host abundance. It must be noted that apart from delaying PUUV infection in MatAb+ individuals^29^,^32^ their high portion may also reduce the infection rate in susceptible individuals through herd immunity^57^. However, further analyses are required to discern whether such an effect accounts for the patterns observed here.

### Implications for human infections

Over the vole density cycles, the high abundances of PUUV-infected bank voles in the winters of increase and peak phases coincided with high rates of registered human NE cases in the region^58^. Interestingly, the winter following the abundance increase in autumn 2004 yielded more NE cases in the region than the winter following the abundance peak in autumn 2005^9^,^58^. Our results demonstrate that despite the lower overall abundance in autumn 2004, the number of infected bank voles was actually higher than in autumn 2005. A contrary situation was observed during the 2007−2009 cycle, where both the abundance of infected bank voles and incidence of human NE infections were higher during the peak- than the increase-phase winter.

Like in our study region, also in a study conducted in the temperate zone, NE incidence mirrored the abundance peaks of PUUV seropositive bank voles^11^. However, an essential difference between the boreal and temperate systems is that, in the temperate zone, the abundance of infected voles and human infections both peak during the summer^11^,^59^,^60^. Although these findings demonstrate that the abundance of PUUV-infected — and presumably infectious — bank voles is the driving force of human NE epidemics, also other factors, such as seasonal changes in human or bank vole behaviour, may play a role in NE epidemiology. For instance, the summer peak in NE incidence in temperate Europe coincides not only with high abundance of PUUV-infected bank voles, but also with higher level of human outdoor activities. In most of Finland, however, summers are times of low NE incidence^9^,^58^, although we demonstrated here that infected bank voles can be highly abundant during the peak phase summers. This contrast could be explained by seasonal change in host behavior: bank voles are known to be attracted to human dwellings in the autumn and winter (especially during unfavourable snow conditions^10^,^61^), and therefore fewer bank vole-human encounters are expected during the summer. Taken together, the epidemiology of boreal NE appears to be mostly regulated by temporal changes in infectious host abundance and seasonal changes in host behaviour, rather than human activities.

In the study region, the lowest incidence of NE were recorded between February and June^9^,^58^, which coincides with the peak infection prevalence but the lowest seasonal abundance of bank voles. Therefore, infection prevalence in the rodent host is not a good measure of human PUUV infection risk, in contrast to what has been suggested for human HCPS incidence in relation to Sin Nombre hantavirus seroprevalence in deer mice^62^,^63^.

## Conclusions

For the first time, patterns of PUUV infection dynamics in cyclic bank voles were studied during the winter when most human NE cases are reported in the boreal zone. We demonstrated that PUUV-infected bank voles are most abundant during the winter of years when the vole population is increasing or peaking. The abundance of infected individuals can be even higher during increasing phases, which may affect the incidence of human NE. PUUV transmission dynamics follow the population dynamics of the host in strikingly regular patterns. We found the seroconversion rate to track the increasing population density in the autumn, supporting a density-dependent transmission pathway. However, in peak density phases, infection prevalence did not respond to the faster summer growth in host abundance but showed a similar temporal pattern to that observed in increasing phases. The higher prevalence of MatAb+ individuals during peak phases may have limited the transmission of PUUV in the population, thus counteracting effects of the increased encounter rate experienced when vole density is high. The overall seroconversion rate was higher outside the breeding season when bank voles are docile and non-territorial, which suggests that aggressive encounters play a minor role in PUUV transmission.

Based on our results, we conclude that PUUV infection dynamics in bank voles are greatly affected by seasonality and cyclicity of vole populations. Therefore, comparisons between cross-sectional studies on hantaviruses in their host species are of little use unless these dynamic factors are controlled for. Only populations sampled in the same season and population growth phase are comparable. Concerning ecological studies of rodent-borne disease agents in general, we urge that trapping effort be extended over several seasons and population densities. We also conclude that PUUV prevalence in bank voles is not a good predictor of NE risk for humans, since its peak in spring co-occurred with the lowest incidence of human cases. Thus far, our study provides the most detailed characterization of PUUV transmission dynamics in bank vole populations. The results provide a solid framework for developing models to predict periods of high human NE risk in boreal Europe, where the disease poses a significant threat to public health.

## Additional Information

**How to cite this article**: Voutilainen, L. *et al.* Temporal dynamics of Puumala hantavirus infection in cyclic populations of bank voles. *Sci. Rep.* **6**, 21323; doi: 10.1038/srep21323 (2016).

## Supplementary Material

Supplementary Figure S1

Supplementary Datasets 2-5

## Figures and Tables

**Table 1:** The known (uppercase) and assigned (lowercase) PUUV status of all bank vole captures on the core grid.

PUUV antibody/infection status	Number
Seronegative; susceptible at *t*	1871
** SS**: negative, captured as negative at *t–1*	**1027**
** **MS: negative, captured as positive at *t–1*	78
** **0S: assigned by weight as not in population at *t–1*	97
** **?S: negative, not captured but assigned by weight as in population at *t–1*	669
** sS**: assigned as susceptible at *t–1*	**555.0**
** **mS: assigned as immune due to maternal antibodies at *t–1*	114.0
Seropositive (P): infected (I/i) or protected by maternal antibodies (M/m) at *t*	1415
** **II: positive, captured as positive at *t–1*	828
** SI**: positive, captured as negative at *t–1*	**223**
** **?P: positive, not captured at *t–1*	364
** **0M: positive year-born, later captured as negative; assigned not in population at *t–1*	47
** **mM: positive year-born, later captured as negative; assigned as immune at *t–1*	31
** **0m: positive year-born, not re-captured as negative; assigned not in population at *t–1*	52
** **mm: positive year-born, not re-captured as negative; assigned as immune by weight	49.6
** **?i: positive adult, or a year-born assigned as infected by weight	184.4
** **ii: assigned as infected and seroconverted before *t–1*	118.2
** si**: assigned as infected and seroconverted between *t–1* and *t*	**59.1**
** **xi: no data to assign PUUV status for *t–1*	7
Total PUUV infection status	
****Susceptible (SS + MS + 0 S + ?S)	1871.0
****Infected (II + SI + ?i)	1235.4
****Maternal antibody positive (0M + mM + 0m + mm)	179.6
Total N of captures	3286

P = seropositive, S/s = susceptible, M/m = maternal antibody positive, I/i = infected, 0 = not in population, ? = unknown, x = undetermined.

Capital letters indicate status deduced from serological history, small letters the assigned status.

In PUUV infection/antibody status, the first character indicates PUUV status at *t−1* (previous trapping session), and second character the PUUV status at *t* (current trapping session).

Seroconversion rates for total population and yearly cohorts per trapping session were calculated from (SI + si)/(SS + SI + ss + si), marked in bold.

**Table 2:** AICc (Akaike information criterion adjusted for sample size) scores of all candidate generalized additive models (GAMs).

Candidate models	Dependent variable		
	Abundance of infected	Infection prevalence	Seroconversion rate
Within-cycle dynamics			
cycle ID + s(cycle month by cycle ID)	**626.4**	543.3	**169.1**
cycle ID + s(cycle month)	646.9	533.2	186.0
s(cycle month)	762.9	**530.1**	184.3
cycle ID	643.9	953.7	193.9
intercept only	761.0	951.2	196.5
Within-year dynamics			
cycle phase + s(month by cycle phase)	**578.1**	507.4	134.6
cycle phase + s(month)	600.1	**490.5**	134.1
s(month)	610.3	497.2	136.5
cycle phase	1374.8	549.0	**132.1**
intercept only	1381.2	550.9	135.5
Variation in relation to breeding season (dichotomous)			
cycle phase * breeding season			130.4
cycle phase + breeding season			**127.9**
breeding season			130.4
cycle phase			130.8
intercept only			134.1
Variation in relation to cohort age and birth year cycle phase			
cycle phase + s(cohort age by cycle phase)		183.3	**156.1**
cycle phase + s(cohort age)		**182.2**	158.3
s(cohort age)		205.3	160.9
cycle phase		519.4	158.7
intercept only		642.2	161.9

s(…) denote GAM smooth terms.

The AICc scores of the best-supported models are written in bold.

**Table 3:** Parameter estimates of the best-supported models analysing the within-cycle and within-year dynamics in the abundance of PUUV infected bank voles, infection prevalence and seroconversion rate.

Dependent variable/source of variation		Within-cycle dynamics			Within-year dynamics		
Abundance of Infected							
*Parametric coefficients*		*Estimate (SE)*	*z*	*P value*	*Estimate (SE)*	*z*	*P value*
intercept		−4.15 (0.24)	−17.1	<0.001	−3.85 (0.15)	−24.9	<0.001
cycle ID	2004–2006	−0.02 (0.25)	−0.1	0.95			
	2007–2009	−0.06 (0.71)	−0.1	0.938			
cycle phase	peak				0.40 (0.17)	2.4	0.016
	low				−2.67 (0.36)	−7.5	<0.001
*Smooth terms*		*Edf (rdf)*	*χ*^*2*^	*P value*	*Edf (rdf)*	*χ*^*2*^	*P value*
cycle month by cycle ID	2001–2003	1 (1)	55	<0.001			
	2004–2006	7.08 (7.08)	190	<0.001			
	2007–2009	5.70 (5.70)	124	<0.001			
month by cycle phase	increase				2.08 (2.08)	13.6	0.001
	peak				2.08 (2.08)	22.7	<0.001
	low				1.95 (1.95)	4.4	0.105
*Random effects*		*σ*^*2*^	*sd*		*σ*^*2*^	*sd*	
site	within cycle 2001–2003	0.64	0.80				
	within cycle 2004–2006	0.31	0.56				
	within cycle 2007–2009	1.34	1.16				
observation					0.70	0.84	
site					0.09	0.31	
Infection prevalence							
*Parametric coefficients*		*Estimate (SE)*	*z*	*P value*	*Estimate (SE)*	*z*	*P value*
intercept		−1.11 (0.15)	−7.3	<0.001	−0.85 (0.22)	−3.8	<0.001
cycle phase	peak	0.19 (0.30)	0.6	0.517			
	low	−0.77 (0.36)	−2.1	0.032			
*Smooth terms*					*Edf (rdf)*	*χ*^*2*^	*P value*
cycle month/month		6.82 (6.82)	41.7	<0.001	3.07 (3.07)	31.1	<0.001
*Random effects*		*σ*^*2*^	*sd*		*σ*^*2*^	*sd*	
site	within cycle 2001–2003	0.75	0.87				
	within cycle 2004–2006	0.98	0.99				
	within cycle 2007–2009	1.42	1.19				
observation					0.43	0.65	
Seroconversion rate							
*Parametric coefficients*		*Estimate (SE)*	*z*	*P value*	*Estimate (SE)*	*z*	*P value*
intercept		−6.06 (1.28)	−4.7	<0.001	−5.31 (0.14)	−37.4	<0.001
cycle ID	2004–2006	0.20 (1.30)	0.2	0.88			
	2007–2009	1.27 (1.85)	0.7	0.497			
cycle phase	peak				−0.27 (0.19)	−1.4	0.161
	low				−2.19 (1.41)	−1.5	0.128
*Smooth terms*		*Edf (rdf)*	*χ*^*2*^	*P value*			
cycle month by cycle ID	2001–2003	1.43 (1.76)	3.8	0.036			
	2004–2006	2.12 (2.71)	2	0.129			
	2007–2009	4.85 (5.53)	3.5	0.007			

Edf = Estimated degrees of freedom; rdf = residual degrees of freedom; σ^2^ = the variance attributable to random effect.

sd = standard deviation of σ^2^.

**Table 4:** Parameter estimates of the best-supported models analysing the PUUV infection prevalence and seroconversion rate during the lifespan of a yearly bank vole cohort.

Dependent variable/source of variation		Parameter estimates		
Infection prevalence				
*Parametric coefficients*		*Estimate (SE)*	*z*	*P value*
intercept		−0.16 (0.13)	−1.2	0.217
birth year cycle phase	peak	−0.27 (0.15)	−1.8	0.08
	low	−3.41 (0.93)	−3.7	< 0.001
*Smooth terms*		*Edf (rdf)*	*χ*^*2*^	*P value*
cohort age		3.89 (4.83)	48.5	<0.001
Seroconversion rate				
*Parametric coefficients*		*Estimate (SE)*	*z*	*P value*
intercept		−5.53 (0.25)	−22.2	< 0.001
birth year cycle phase	peak	−0.11 (0.30)	−0.4	0.716
	low	−2.12 (1.70)	−1.2	0.219
*Smooth terms*		*Edf (rdf)*	*χ*^*2*^	*P value*
cohort age by birth year cycle phase	increase	3.62 (4.49)	1.8	0.125
	peak	1.00 (1.00)	0.1	0.714
	low	1.00 (1.00)	0.9	0.358

Edf = Estimated degrees of freedom; rdf = Residual degrees of freedom.
