# Erratum: Temporal dynamics of Puumala hantavirus infection in cyclic populations of bank voles

**DOI:** 10.1038/srep23853

**Published:** 2016-04-13

**Authors:** Liina Voutilainen, Eva R. Kallio, Jukka Niemimaa, Olli Vapalahti, Heikki Henttonen

Scientific Reports 6: Article number: 2132310.1038/srep21323; published online: 02182016; updated: 04132016

Supplementary Datasets 2–5 were inadvertently published with the original version of this Article. These have now been removed.

