# Supplementary material for: Temporal dynamics of Puumala hantavirus infection in cyclic populations of bank voles
Source: Sci Rep. 2016 Feb 18;6:21323. doi: 10.1038/srep21323 (PMC4758042; doi:10.1038/srep21323)
Supplement: Supplementary Figure S1 [file srep21323-s1.doc]

**Temporal dynamics of Puumala hantavirus infection in cyclic populations of bank voles**

Liina Voutilainen, Eva R. Kallio, Jukka Niemimaa, Olli Vapalahti, and Heikki Henttonen


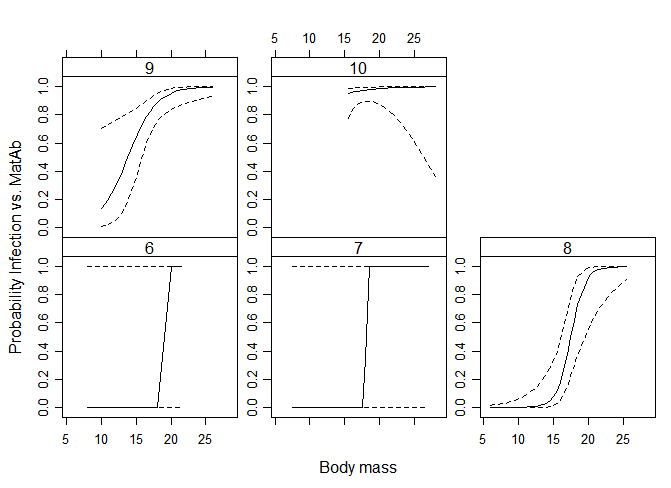


**Supplementary Figure S1. Determining the probability of genuine PUUV infection as opposed to MatAb.** Predicted probabilities of a seropositive year-born individual being genuinely PUUV infected as opposed to carrying maternal antibodies in relation to body mass (grams) and month (June to October).
